# Control and Elimination of Extensively Drug-Resistant *Acinetobacter baumanii* in an Intensive Care Unit 

**DOI:** 10.3201/eid2510.181626

**Published:** 2019-10

**Authors:** Amanda Chamieh, Tania Dagher Nawfal, Tala Ballouz, Claude Afif, George Juvelekian, Sani Hlais, Jean-Marc Rolain, Eid Azar

**Affiliations:** University of Balamand, Beirut, Lebanon (A. Chamieh, T. Ballouz, C. Afif, G. Juvelekian, E. Azar);; Aix-Marseille University, Marseille, France (T.D. Nawfal, J.-M. Rolain);; Saint Joseph University and American University of Beirut, Beirut (S. Hlais)

**Keywords:** XDR, MDR, Acinetobacter baumanii, carbapenem, antimicrobial stewardship, antimicrobial resistance, bacteria, Lebanon, France

## Abstract

We decreased antimicrobial drug consumption in an intensive care unit in Lebanon by changing to colistin monotherapy for extensively drug-resistant *Acinetobacter baumanii* infections. We saw a 78% decrease of *A. baumanii* in sputum and near-elimination of *bla*_oxa-23_-carrying sequence type 2 clone over the 1-year study. Non–*A. baumanii* multidrug-resistant infections remained stable.

The antimicrobial stewardship program (ASP) at Saint Georges Hospital University Medical Center (SGHUMC), a 400-bed tertiary-care center in Beirut, Lebanon, requires an infectious disease (ID) specialist to preauthorize use of restricted broad-spectrum antimicrobial drugs. The ASP regularly monitors the rate of nosocomial infections and the total hospital antimicrobial drug consumption. In the first quarter of 2015, the incidence of extensively drug-resistant (XDR) *Acinetobacter baumanii* bloodstream infections reached its highest level, 0.47/1,000 patient-days (*1*). Since 2012, the monthly carbapenem consumption increased steadily, reaching 130 defined daily doses (DDD)/1,000 patient-days in 2015, an absolute increase of 30 DDD/1,000 patient-days during that time. Severely ill patients with predisposing conditions are more likely to develop difficult-to-treat *A. baumanii* infections. Despite the existing controversy, this patient population routinely is treated with a carbapenem/colistin combination (*2**–**8*). 

We evaluated 100 nonduplicate XDR *A. baumanii* isolates at SGHUMC and found no synergy between colistin and carbapenem by the checkerboard technique (*9*). Consequently, SGHUMC withdrew combination therapy for XDR *A. baumanii* infections. Our aim was to evaluate the effect of a carbapenem-sparing regimen on ICU antimicrobial consumption, clinical outcome, and microbiological flora.

## The Study

The ASP, ID team, and intensive care unit (ICU) physicians approved a plan to reduce use of empiric carbapenems in the ICU and use colistin, tigecycline, or both for patients confirmed with or at high risk for *A. baumanii* infections. ID physicians evaluated the clinical severity and hemodynamic stability of each patient and had final discretion to prescribe either colistin or tigecycline.

We included all ICU admissions in the study, even recurrent admissions. This ICU has a multidrug-resistant organism surveillance program that collects a sputum sample for culture every third day for intubated patients with abundant secretions. We used these cultures for our evaluation. We considered any culture sample outside this practice a duplicate and excluded it from our analysis. During the study period, we did not modify infection control practices. The study was approved by the institutional review board of SGHUMC. 

We retrieved data from the hospital’s computerized ordering system and examined medical records of all ICU admissions during February 1, 2016–January 31, 2017. Clinical data included patient demographics, admission diagnosis, and presence of mechanical ventilation. During February 1–June 30, 2016 (period 1), patients received colistin/carbapenem combination therapy for *A. baumanii* infections. During July 1, 2016–January 31, 2017 (period 2), we applied our intervention. We recorded the total number of bacterial cultures collected from the ICU and noted the site and date of sampling. 

We considered the isolation density the number of clinical isolates/1,000 patient-days and the rate of ventilator-associated pneumonia (VAP) the number of VAP events/1,000 patient-days. We defined variables according to guidelines for XDR *A. baumanii* from the US Centers for Disease Control and Prevention and World Health Organization (*10*). We calculated case-fatality and VAP rates following guidelines from the American Thoracic Society and Infectious Diseases Society of America (*11*). 

We grouped antimicrobial drugs into 5 categories: group 1, antimicrobial drugs that do not require ID preapproval, such as third-generation cephalosporins, amoxicillin/clavulanic acid, and quinolones; group 2, oral vancomycin and metronidazole used for *Clostridioides difficile* therapy; group 3, imipenem and meropenem; group 4, broad-spectrum carbapenem-sparing regimens, including piperacillin/tazobactam, cefepime, ceftazidime, amikacin; and group 5, the XDR *A. baumanii*–active antimicrobial drugs colistin and tigecycline. We measured antimicrobial drug consumption by DDD per 1,000 patient-days (Table 1).

**Table 1:** Patient demographics, VAP incidence, treatment courses, and antimicrobial drug consumption in study of carbapenem-sparing regimen for XDR *Acinetobacter baumannii* in an ICU, Beirut, Lebanon*

Characteristics	Period 1†	Period 2‡	p value	
Patient data				
No. patients	213	324	NA	
Sex				
F	79	144		
M	134	180		
Mean age	69	68		
Mean length of hospital stay, d	6.8	6		
Days in ICU	1,128	1,804		
Type of admission				
Medical	163	253	NA	
Surgical	50	71		
Admitted from home or ED	73	114		
Transferred from ward	79	141		
Transferred from other hospital	10	16		
Postoperative	51	57		
Intubation				
At admission	64	85		
After admission	14	16		
Outcome				
*A. baumanii* VAP incidence, %	15	3.7	0.007	
Discharged	170	259		
Deceased	43	64		
Total AB VAP events	32	12		
Deceased during VAP	17	4		
ICU mean mortality rate/month, %	20.4	19.3	0.168	
AB VAP case fatality ratio, %	7.9	1.2	0.006	
No. XDR *A. baumanii* VAP courses received				
Colistin and carbapenem	17	2		
Colistin and tigecycline	6	2		
Colistin monotherapy	6	6		
Tigecycline	3	2		
Carbapenem consumption, DDD§				
Group 1	333	320	0.465	
Group 2	455	224	**0.042**	
Group 3	541	223	**<0.005**	
Group 4	165	145	0.808	
Group 5				
Colistin	20	9	**<0.019**	
Tigecycline	84	62	0.570	
Total restricted antimicrobial drugs, DDD	1,265	663	**<0.005**	

*Bold indicates statistical significance. DDD, defined daily doses; ED, emergency department, ESBL, extended-spectrum β-lactamase; ICU, intensive care unit; NA, not applicable; VAP, ventilator-associated pneumonia; XDR, extensively drug-resistant.  †During February 1, 2016–June 30, 2016, ICU patients received colistin/carbapenem therapy for *A. baumannii* infections.  ‡During July 1, 2016–January 31, 2017, ICU implemented carbapenem-sparing regimen for *A. baumannii* infections. §Group 1, antimicrobial drugs that do not require infectious disease specialist preapproval, such as third-generation cephalosporins, amoxicillin/clavulanic acid, and quinolones; group 2, oral vancomycin and metronidazole used for *Clostridioides difficile* therapy; group 3, imipenem and meropenem; group 4, broad-spectrum carbapenem-sparing regimens, including piperacillin/tazobactam, cefepime, ceftazidime, amikacin; and group 5, the XDR A. baumanii–active antimicrobial drugs colistin and tigecycline.

We sent 48 laboratory-confirmed *A. baumanii* isolates, 31 collected during period 1 and 17 during period 2, to IHU-Méditerranée Infection, Aix-Marseille, France, for testing. Samples underwent 4 types of testing: matrix-assisted laser desorption/ionization time-of-flight mass spectrometry (Microflex; Bruker Daltonics, https://www.bruker.com); antimicrobial susceptibility testing by disk diffusion method and interpreted according to the European Committee of Antimicrobial Susceptibility Testing 2017; real-time PCR to screen for carbapenemase-encoding genes; and multilocus sequence typing to determine genetic relationships among the isolates.

The ICU admitted 536 patients during the study period; 3 were readmissions. Patient characteristics between the 2 periods were statistically similar (Table 1). Throughout the study, the incidence of *A. baumanii* VAP decreased from 154.9 to 38/1,000 patient-days (p = 0.007) and the *A. baumanii* VAP case-fatality ratio dropped from 79 to 12/1,000 patient-days. Non–*A. baumanii* VAP incidence decreased from 62 to 51/1,000 patient-days. ICU mortality rates from all causes remained unchanged between period 1 and period 2 (Table 1).

**Figure 1:**
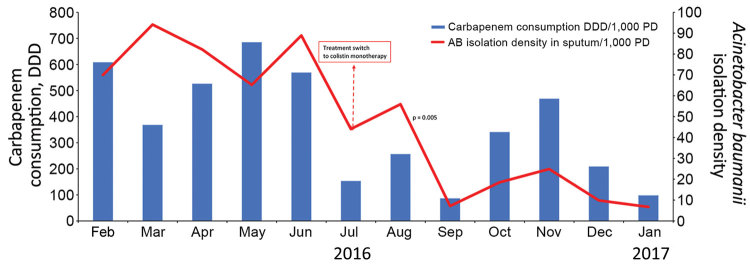
Isolation density of *Acinetobacter baumanii* in sputum cultures versus carbapenem consumption in the intensive care unit (ICU) of Saint Georges Hospital University Medical Center, Beirut, Lebanon, during February 1, 2016–January 31, 2017. Rates are measured per 1,000 patient-days. Dashed arrow represents the beginning of period 2 in which we implemented a carbapenem-sparing regimen. DDD, defined daily dose; PD, patient days.

Consumption of group 1 and group 4 antimicrobial drugs was statistically similar during the 2 periods (Table 1). Carbapenem consumption decreased by 59%, a total of 318 DDD/1,000 patient-days, and overall restricted antimicrobial drug consumption dropped 637 DDD/1,000 patient-days (p<0.005). Because isolation of *A. baumanii* decreased substantially, colistin consumption also decreased by 55%, from 20 DDD/1,000 patient-days in period 1 to 9 DDD/1,000 patient-days in period 2 (p = 0.019) (Figure 1). Tigecycline consumption remained statistically unchanged (84 DDD/1,000 patient-days in period 1, 62 DDD/1,000 patient-days in period 2). Of note, group 2 *C. difficile* therapy consumption dropped by 231 DDD/1,000 patient-days (p = 0.042), a 51% decrease that likely mirrors reduction in *C. difficile* infections.

**Figure 2:**
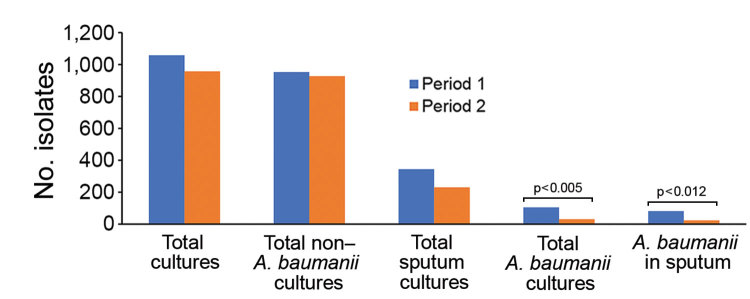
Isolation density of *Acinetobacter baumanii* and non–*A. baumanii* in the intensive care unit (ICU) of Saint Georges Hospital University Medical Center, Beirut, Lebanon, during February 1, 2016–January 31, 2017. Rates are measured in 1,000 patient-days. During period 1, February 1–June 31, 2016, ICU patients received colistin/carbapenem combination therapy for *A. baumanii*. During period 2, July 1, 2016–January 31, 2017, we implemented a carbapenem-sparing regimen in the ICU.

The *A. baumanii* isolate density in sputum cultures decreased by 70.7%, from 82 to 24/1,000 patient-days, positively correlating with the fall in carbapenem consumption (p = 0.004) (Figure 1). The number of non–*A. baumanii* multidrug-resistant (MDR) isolates did not increase (Figure 2).

All 48 *A. baumanii* isolates carried extended-spectrum β-lactamase *bla*_TEM-1_ genes. The 31 isolates from period 1 were XDR; 30 carried the class D carbapenemase *bla*_oxa-23_ gene, and 1 carried the *bla*_oxa-24_ gene. Multilocus sequence typing revealed 3 sequence types (STs) in period 1: ST2, 29/31 (93.5%); ST699, 1/31 (3%); and ST627, 1/31 (3%) (Table 2). In period 2, *A. baumanii* ST2 disappeared; 58.8% (10/17) of isolates belonged to ST25 and 5.9% (1/17) belonged to ST99. The remainder belonged to 6 new STs, assigned ST1200, 1201, 1202, 1203, 1204, and 1205 (35.2%). Of the 17 isolates from period 2, 6 carried the *bla*_oxa-23_ gene, 5 the *bla*_oxa-24_ gene, and 3 both genes. 

**Table 2:** Specimen type, site of collection, and microbiologic characteristics in study of carbapenem-sparing regimen for extensively drug-resistant *Acinetobacter baumannii* in an ICU, Beirut, Lebanon*

Specimens and testing	Period 1†	Period 2‡
Specimen type, no.		
Sputum	31	11
Blood	0	3
Wound site or catheter tip	0	3
Site of collection, no.		
Intensive care unit	21	12
Regular floor	10	5
Total no.	31	17

Antimicrobial drug susceptibility testing by disc diffusion, %		
Cefepime/ceftazidime	0	**64.7**
Piperacillin/tazobactam	0	**17.65**
Imipenem	0	**17.65**
Colistin	100	100
Total extensively drug-resistant	100	**35.3**

Carbapenemase genes, no. (%)§		
ESBL *bla*_TEM-1_	31 (100)	17 (100)
*bla*_oxa-23_	30 (96.8)	6 (35.3)
*bla*_oxa-24_	1 (3.2)	5 (29.4)
*bla*_oxa-23_ and *bla*_oxa-24_	0	3 (17.6)

Sequence type, no. (%)¶		
ST2	**29 (93.5)**	**0**
ST699	**1 (3.25)**	0
ST627	**1 (3.25)**	0
ST25	0	**10 (58.9)**
ST99	0	**1 (5.8)**
New STs, 1200–1206	0	**6 (35.3)**

*Bold indicates statistical significance. ESBL, extended-spectrum beta-lactamase; ICU, intensive care unit; MLST, multilocus sequence typing; PCR, polymerase chain reaction; ST, sequence type.  †During February 1, 2016–June 30, 2016, ICU patients received colistin/carbapenem therapy for *A. baumannii* infections.  ‡During July 1, 2016–January 31, 2017, ICU implemented carbapenem-sparing regimen for *A. baumannii* infections. §Determined by PCR. ¶Determined by multilocus sequence typing.

Overall, XDR *A. baumanii* isolation decreased by 64.7% from period 1 to period 2. In addition, isolates from period 2 were more antimicrobial-susceptible than in period 1: 64.8% (11/17) sensitive to ceftazidime and cefepime, 17.6% (3/17) to piperacillin/tazobactam, and 17.6% (3/17) to carbapenems (Table 2).

## Conclusions

Our prudent use of antimicrobial drugs did not increase mortality rates and had a dramatic effect on antimicrobial consumption and MDR *A. baumanii* isolate density. A longer study period and larger sample likely would reveal additional effects on XDR infections and outcomes. Many factors could have affected the study results, including patient referrals and seasonality. However, the microbiological findings strongly point to high rates of carbapenem consumption as a sustaining factor in survival of XDR *A. baumanii* ST2 in our facility. By reducing carbapenem consumption, we broke a vicious cycle.

In the era where clinicians must manage severely ill, MDR-colonized patients, relying on existing guidelines is not enough. A creative, multidisciplinary approach with knowledge of local epidemiology is key to controlling MDR and XDR infections. Investing time in accurate diagnosis and implementing targeted carbapenem-sparing strategies for initial treatment is only possible through trusted collaboration between ID and ICU physicians. The dedication of the ASP and microbiology departments at this facility is an example of a successful active surveillance program for antimicrobial drug consumption and resistance profiles, especially when developing standards of care tailored to meet an institution’s needs.

## References

[1] Ballouz T, Aridi J, Afif C, Irani J, Lakis C, Nasreddine R, et al. Risk factors, clinical presentation, and outcome of *Acinetobacter baumannii* bacteremia. Front Cell Infect Microbiol. 2017;7:156. 10.3389/fcimb.2017.00156 28523249 PMC5415554

[2] Falagas ME, Rafailidis PI, Ioannidou E, Alexiou VG, Matthaiou DK, Karageorgopoulos DE, et al. Colistin therapy for microbiologically documented multidrug-resistant Gram-negative bacterial infections: a retrospective cohort study of 258 patients. Int J Antimicrob Agents. 2010;35:194–9. 10.1016/j.ijantimicag.2009.10.005 20006471

[3] Batirel A, Balkan II, Karabay O, Agalar C, Akalin S, Alici O, et al. Comparison of colistin-carbapenem, colistin-sulbactam, and colistin plus other antibacterial agents for the treatment of extremely drug-resistant *Acinetobacter baumannii* bloodstream infections. Eur J Clin Microbiol Infect Dis. 2014;33:1311–22. 10.1007/s10096-014-2070-6 24532009

[4] Cai Y, Chai D, Wang R, Liang B, Bai N. Colistin resistance of *Acinetobacter baumannii*: clinical reports, mechanisms and antimicrobial strategies. J Antimicrob Chemother. 2012;67:1607–15. 10.1093/jac/dks084 22441575

[5] Haddad FA, Van Horn K, Carbonaro C, Aguero-Rosenfeld M, Wormser GP. Evaluation of antibiotic combinations against multidrug-resistant *Acinetobacter baumannii* using the E-test. Eur J Clin Microbiol Infect Dis. 2005;24:577–9. 10.1007/s10096-005-1366-y 16133416

[6] Rigatto MH, Vieira FJ, Antochevis LC, Behle TF, Lopes NT, Zavascki AP. Polymyxin B in combination with antimicrobials lacking in vitro activity versus polymyxin B in monotherapy in critically ill patients with *Acinetobacter baumannii* or *Pseudomonas aeruginosa* infections. Antimicrob Agents Chemother. 2015;59:6575–80. 10.1128/AAC.00494-15 26259799 PMC4576098

[7] Tripodi MF, Durante-Mangoni E, Fortunato R, Utili R, Zarrilli R. Comparative activities of colistin, rifampicin, imipenem and sulbactam/ampicillin alone or in combination against epidemic multidrug-resistant *Acinetobacter baumannii* isolates producing OXA-58 carbapenemases. Int J Antimicrob Agents. 2007;30:537–40. 10.1016/j.ijantimicag.2007.07.007 17851050

[8] Liu X, Zhao M, Chen Y, Bian X, Li Y, Shi J, et al. Synergistic killing by meropenem and colistin combination of carbapenem-resistant *Acinetobacter baumannii* isolates from Chinese patients in an in vitro pharmacokinetic/pharmacodynamic model. Int J Antimicrob Agents. 2016;48:559–63. 10.1016/j.ijantimicag.2016.07.018 27670371

[9] Hajjar Soudeiha M, Dahdouh E, Daoud Z, Sarkis DK. Phenotypic and genotypic detection of β-lactamases in *Acinetobacter* spp. isolates recovered from Lebanese patients over a 1-year period. J Glob Antimicrob Resist. 2018;12:107–12. 10.1016/j.jgar.2017.09.016 28986323

[10] Magiorakos AP, Srinivasan A, Carey RB, Carmeli Y, Falagas ME, Giske CG, et al. Multidrug-resistant, extensively drug-resistant and pandrug-resistant bacteria: an international expert proposal for interim standard definitions for acquired resistance. Clin Microbiol Infect. 2012;18:268–81. 10.1111/j.1469-0691.2011.03570.x 21793988

[11] Kalil AC, Metersky ML, Klompas M, Muscedere J, Sweeney DA, Palmer LB, et al. Management of adults with hospital-acquired and ventilator-associated pneumonia: 2016 clinical practice guidelines by the Infectious Diseases Society of America and the American Thoracic Society. Clin Infect Dis. 2016;63:e61–111. 10.1093/cid/ciw353 27418577 PMC4981759

